# Genome-wide characterization of the tomato GASA family identifies SlGASA1 as a repressor of fruit ripening

**DOI:** 10.1093/hr/uhac222

**Published:** 2022-09-28

**Authors:** Dan Su, Kaidong Liu, Zhuoshu Yu, Ying Li, Yaoxin Zhang, Yunqi Zhu, Yi Wu, Hongyu He, Xiaodan Zeng, Honglin Chen, Don Grierson, Heng Deng, Mingchun Liu

**Affiliations:** Key Laboratory of Bio-Resource and Eco-Environment of Ministry of Education, College of Life Sciences, Sichuan University, Chengdu, 610065, Sichuan, China; Life Science and Technology School, Lingnan Normal University, Zhanjiang, 524048, China; Key Laboratory of Bio-Resource and Eco-Environment of Ministry of Education, College of Life Sciences, Sichuan University, Chengdu, 610065, Sichuan, China; Key Laboratory of Bio-Resource and Eco-Environment of Ministry of Education, College of Life Sciences, Sichuan University, Chengdu, 610065, Sichuan, China; Key Laboratory of Bio-Resource and Eco-Environment of Ministry of Education, College of Life Sciences, Sichuan University, Chengdu, 610065, Sichuan, China; Key Laboratory of Bio-Resource and Eco-Environment of Ministry of Education, College of Life Sciences, Sichuan University, Chengdu, 610065, Sichuan, China; Key Laboratory of Bio-Resource and Eco-Environment of Ministry of Education, College of Life Sciences, Sichuan University, Chengdu, 610065, Sichuan, China; Institute of Agro-Products Processing Science and Technology, Sichuan Academy of Agricultural Sciences, Chengdu, 610066, China; Institute of Agro-Products Processing Science and Technology, Sichuan Academy of Agricultural Sciences, Chengdu, 610066, China; Institute of Agro-Products Processing Science and Technology, Sichuan Academy of Agricultural Sciences, Chengdu, 610066, China; School of Biosciences, University of Nottingham, Sutton Bonington Campus, Loughborough, LE12 5RD, United Kingdom; Key Laboratory of Bio-Resource and Eco-Environment of Ministry of Education, College of Life Sciences, Sichuan University, Chengdu, 610065, Sichuan, China; Key Laboratory of Bio-Resource and Eco-Environment of Ministry of Education, College of Life Sciences, Sichuan University, Chengdu, 610065, Sichuan, China

## Abstract

Gibberellins (GAs) play crucial roles in a wide range of developmental processes and stress responses in plants. However, the roles of GA-responsive genes in tomato (*Solanum lycopersicum*) fruit development remain largely unknown. Here, we identify 17 *GASA* (*Gibberellic Acid-Stimulated Arabidopsis*) family genes in tomato. These genes encode proteins with a cleavable signal peptide at their N terminus and a conserved GASA domain at their C terminus. The expression levels of all tomato *GASA* family genes were responsive to exogenous GA treatment, but adding ethylene eliminated this effect. Comprehensive expression profiling of *SlGASA* family genes showed that *SlGASA1* follows a ripening-associated expression pattern, with low expression levels during fruit ripening, suggesting it plays a negative role in regulating ripening. Overexpressing *SlGASA1* using a ripening-specific promoter delayed the onset of fruit ripening, whereas *SlGASA1*-knockdown fruits displayed accelerated ripening. Consistent with their delayed ripening, *SlGASA1*-overexpressing fruits showed significantly reduced ethylene production and carotenoid contents compared to the wild type. Moreover, ripening-related genes were downregulated in *SlGASA1*-overexpressing fruits but upregulated in *SlGASA1*-knockdown fruits compared to the wild type. Yeast two-hybrid, co-immunoprecipitation, transactivation, and DNA pull-down assays indicated that SlGASA1 interacts with the key ripening regulator FRUITFULL1 and represses its activation of the ethylene biosynthesis genes *ACS2* and *ACO1*. Our findings shed new light on the role and mode of action of a GA-responsive gene in tomato fruit ripening.

## Introduction

Gibberellins (GAs) are a class of tetracyclic diterpenoid phytohormones that regulate diverse aspects of plant development and stress responses, including stem elongation, cell division, seed germination, trichome formation, lateral root formation, flower and fruit development, and resistance to both biotic and abiotic stress [1–8]. GA signaling is based on the E3 ubiquitin ligase-mediated polyubiquitination and subsequent proteolytic degradation of inhibitory DELLA proteins [2]. When bioactive GA binds to the GA receptor GID1 (GIBBERELLIN-INSENSITIVE DWARF1), the activated receptor binds to DELLA proteins and enables their polyubiquitylation by the ubiquitin ligase E3 SKP1–CULLIN–F-box (SCF) complex and their subsequent degradation via 26S proteasome-mediated proteolysis. The proteolysis of DELLAs releases their transcription factor partners from inhibition and triggers the transcriptional reprogramming of GA-responsive genes [2].

The GA-responsive gene *GAST1* (*Gibberellin Stimulated Transcript 1*), which was cloned from the GA-deficient tomato (*Solanum lycopersicum*) mutant *gib1*, was the first *GASA* (*Gibberellic Acid-Stimulated Arabidopsis*) family gene identified in plants [9]. *GASA* family genes encode small polypeptides that possess three domains: an N-terminal putative signal peptide consisting of 18–29 amino acids, a highly variable region with polar amino acid residues in the middle of the protein sequence, and a 60-amino acid C-terminal domain with 12 conserved cysteine residues (GASA domain) that is thought to be essential for protein stability [10–13]. To date, *GASA* family genes have been identified in plant species including Arabidopsis (*Arabidopsis thaliana*) [11, 14, 15], potato (*Solanum tuberosum*) [5, 6, 8], Transvaal daisy (*Gerbera hybrida*) [16], petunia (*Petunia hybrida*) [17], rice (*Oryza sativa*) [18, 19], strawberry (*Fragaria* × *ananassa*) [20, 21], maize (*Zea mays*) [22], apple (*Malus domestica*) [12], rubber tree (*Hevea brasiliensis*) [23], wheat (*Triticum aestivum*) [24], soybean (*Glycine max*) [25], grapevine (*Vitis vinifera*) [13], *Populus* [26], and tobacco (*Nicotiana tabacum*) [27]. GASA family genes are involved in various developmental and physiological processes in different plants, such as seed development [13, 28], flowering [12, 28, 29], lateral root formation [15, 22], cell elongation [16, 20, 21], fruit development [21], and responses to biotic and abiotic stress [23, 30, 31]. Although tomato *GAST1* was the first GA-regulated *GASA* family gene identified in plants [9], its physiological role in tomato remains unclear. Moreover, very little is known about tomato *GASA* family genes and their functions.

Tomato is not only an important cash crop but also a model plant for studying fruit development and climacteric ripening [32]. Using tomato as a model, ethylene was shown to play an essential role in controlling climacteric fruit ripening [33, 34]. In addition to ethylene, several transcription factors such as RIPENING INHIBITOR (RIN), NON-RIPENING (NOR), FRUITFULL1/2 (FUL1/2), APETALA2a (AP2a), and TOMATO AGAMOUS-LIKE 1 (TAGL1) act as key regulators of ripening [33, 35–39]. Interestingly, GA was recently shown to play a negative role in tomato fruit ripening in an ethylene-dependent manner [40]. Nevertheless, the roles of GA-responsive genes in tomato fruit development and ripening remain elusive.

Here, by identifying and analysing members of the tomato *GASA* gene family, a family known to be responsive to GA, we revealed that SlGASA1, a member of the SlGASA family, plays a negative role in fruit ripening by repressing the effect of FUL1 on regulating genes involved in ethylene biosynthesis. Our study provides new insights into the role of this GA-responsive gene in the regulatory network controlling fruit ripening.

## Results

### Identification of 17 *GASA* family genes in the tomato genome

We identified 17 *GASA* genes in the whole tomato genome (SL4.0) using Hmmsearch and multi-sequence alignment based on the protein sequences of GASA family members in Arabidopsis. All GASAs contain a signal peptide of 18–29 amino acids at their N terminus, a variable region with polar amino acid residues in the middle of the protein sequence, and a GASA domain (PF02704) at the C terminus (Fig. S1, see online supplementary material). In addition to *SlGASA1* (also known as *GAST1*, as reported in 1992 [9]), the 16 other *SlGASA* family genes were named based on their locations on the tomato chromosomes. Detailed information about these genes/proteins, including their chromosomal coordinates, isoelectric points, molecular weights, protein lengths, and open reading frame (ORF) lengths, is provided in Table S1 (see online supplementary material).

**Figure 1:**
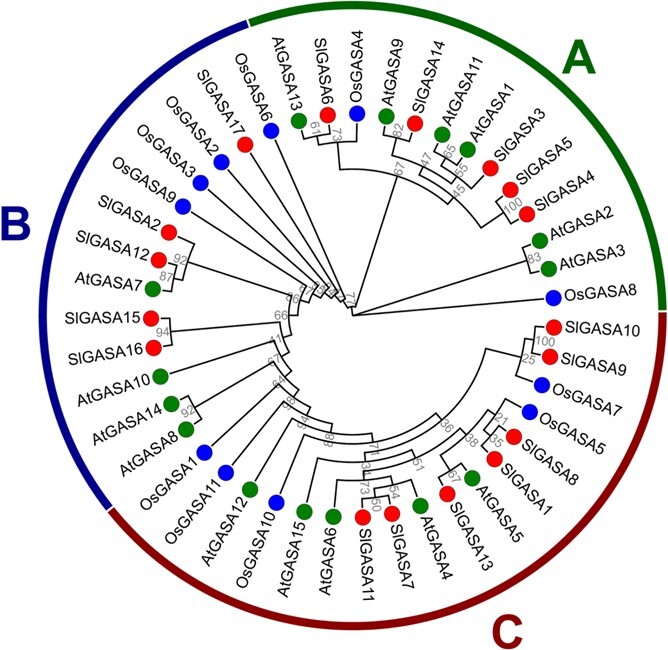
Maximum likelihood tree of GASAs in *Arabidopsis thaliana*, *Solanum lycopersicum*, and *Oryza sativa*. All family members were divided into three clades, which are represented by different colors. Proteins from different species are labeled with colored dots. The bootstrap values are shown at each branch.

To investigate the phylogenetic relationships among GASA proteins, we used the protein sequences of 15 AtGASAs from Arabidopsis, 11 OsGASAs in rice (*O. sativa*), and 17 SlGASAs in tomato to construct a maximum-likelihood (ML) tree. All GASAs were grouped into three clades, as previously reported in Arabidopsis [11, 19] and other plant species [8, 12, 22, 24, 25, 27]. Clade A contains five SlGASAs, six AtGASAs, and two OsGASAs. Clade B consists of five SlGASAs, four AtGASAs, and four OsGASAs. Clade C comprises seven SlGASAs, along with five AtGASAs, and five OsGASAs (Fig. 1).

### Analysis of the conserved domains and promoter sequences of tomato GASA/*GASA* family members

To further investigate the diversity of SlGASA protein structure and to predict their putative functions, we identified their conserved domains and motifs based on searches of the Pfam and MEME databases. As shown in Fig. S2 (see online supplementary material), all SlGASA family members harbor the GASA domain and three conserved motifs (i.e. motif 1, motif 2, and motif 3). Motif 3 is linked to the conserved signal peptide of the GASA family, while motifs 1 and 2 form the GASA domain.

To predict the potential roles of *SlGASA* family genes in different developmental steps, we predicted the presence of *cis*-acting elements in their promoter regions using the PlantCARE database. We identified *cis*-acting elements which are related to plant development, abiotic and biotic stresses, and plant hormone responsiveness (Data S1, see online supplementary material). Interestingly, we only detected the typical GA-responsive element (TATC-box) in the promoter of SlGASA1 but not in that of other members of the SlGASA family, pointing to a potential role for SlGASA1 in GA-mediated processes (Data S1, see online supplementary material).

### Responsiveness of *SlGASA* family gene expression to exogenous GA treatment

**Figure 2:**
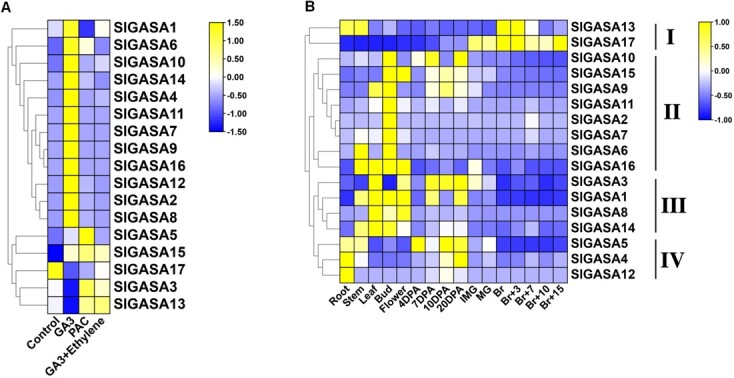
GA-responsiveness and expression profiles of tomato *GASA* family genes. **A** Heatmap representation of the responsiveness of *SlGASA* family genes treated with GA_3_, PAC (paclobutrazol), and GA_3_ + ethylene. RT-qPCR was conducted after treatment, as described in the ‘Materials and methods’ section. The scale (−1.5 to +1.5) represents the expression level (from low to high). **B** Heatmap representation of the relative expression of *SlGASA*s in different tomato tissues and developmental stages. 4 to 20 DPA: the fruit at stages from 4 to 20 days after anthesis; Br: fruit at breaker stage; Br + 3 to Br + 15: the fruits at the stages from 3 to 15 days post-breaker stage; IMG: fruit at immature green stage; MG: fruit at mature green stage. The scale (−1 to +1) represents the expression level (from low to high).

Since *SlGASA1* was first identified as a GA-inducible gene, we assessed the responsiveness of *SlGASA* family genes to treatment with GA_3_ and the GA biosynthesis inhibitor paclobutrazol (PAC) via reverse transcription quantitative PCR (RT-qPCR). The transcript levels of most *SlGASA* genes (SlGASA1*, SlGASA6, SlGASA10, SlGASA14, SlGASA4, SlGASA11, SlGASA7, SlGASA9, SlGASA16, SlGASA12, SlGASA2, SlGASA8, SlGASA5,* and *SlGASA15*) were upregulated by GA_3_ treatment (Fig. 2A; Fig. S3, see online supplementary material). Among these genes, only *SlGASA1* expression was repressed by PAC treatment (Fig. 2A; Fig. S3, see online supplementary material). However, *SlGASA3* and *SlGASA13* expression was repressed by GA_3_ and activated by PAC treatment (Fig. 2A; Fig. S3, see online supplementary material). Interestingly, the application of exogenous ethylene to GA_3_-treated fruits eliminated the effect of GA_3_ on *SlGASA* transcript levels (Fig. 2A; Fig. S3, see online supplementary material). These results suggest that all *SlGASA* family members are sensitive to exogenous GA treatment and that ethylene can repress this GA responsiveness in tomato.

### Expression profiles of *SlGASA*s in various tissues and developmental stages

To investigate the potential functions of *SlGASA* family genes in tomato, we performed RT-qPCR to examine the accumulation of *SlGASA* transcripts in various tissues and different developmental stages (including root, stem, leaf, bud, flower tissue, and 11 fruit tissues at different developmental and ripening stages). As shown in Fig. 2B, the 17 Sl*GASA* family genes can be divided into four subgroups based on their expression patterns during plant growth and fruit development. Subgroup I consists of two *SlGASA* genes (*SlGASA13* and *SlGASA17*) whose transcript accumulation peaks at the breaker (Br) stage. Subgroup II contains eight genes (*SlGASA10*, *15*, *9*, *11*, *2*, *7*, *6*, and *16*) with preferential expression in flower buds (Fig. 2B; Fig. S4, see online supplementary material), suggesting a putative role in flower development and fruit set. Genes from subgroup III (*SlGASA3, 1, 8, 14*) show transcript accumulation primarily in vegetative tissue and unripe fruits (Fig. 2B; Fig. S4, see online supplementary material), suggesting roles in plant growth and fruit development. Subgroup IV genes (*SlGASA5, 4, 12*) are expressed at the highest levels in roots and young fruits (Fig. 2B; Fig. S4, see online supplementary material).

Interestingly, among all *SlGASA* family members, *SlGASA1*, *SlGASA3*, and *SlGASA5* transcripts declined in abundance after the breaker stage (Fig. 2B; Fig. S4, see online supplementary material), suggesting that these genes play negative roles in fruit ripening. We mined public transcriptome deep sequencing (RNA-seq) data [41] to validate our results. Based on this analysis, *SlGASA13* and *SlGASA17* transcripts accumulated during fruit ripening, while most other *SlGASA* genes, especially *SlGASA1, SlGASA3,* and *SlGASA5*, exhibited decreased expression during fruit ripening (Fig. S5, see online supplementary material). These expression profiles support the notion that *SlGASA* family genes are involved in fruit ripening.

### Subcellular localization of SlGASA1 and its responsiveness to ethylene

**Figure 3:**
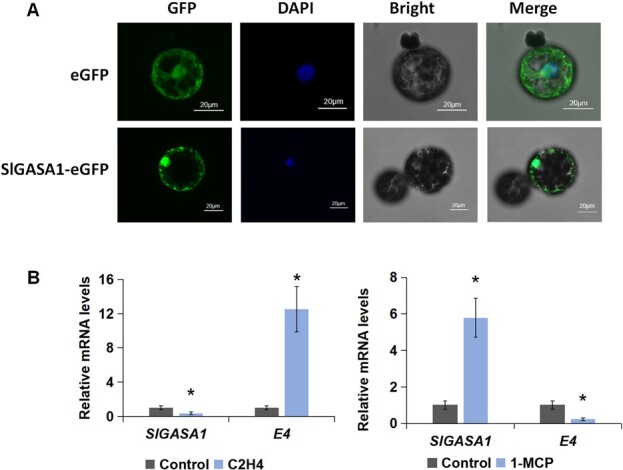
Subcellular localization of SlGASA1 and responsiveness of *SlGASA1* to ethylene. **A** Subcellular localization of the SlGASA1-GFP fusion protein. Protoplasts were obtained from *N. benthamiana* leaves; confocal micrographs show the localization of eGFP (control) and SlGAST1-eGFP. **B** RT-qPCR analysis of *SlGAST1* relative expression levels in WT fruits treated with ethylene or 1-MCP. ^*^P < 0.05 (Student’s *t*-test).

GAs were recently shown to play a negative role in fruit ripening [40]. To investigate the functional significance of GA-responsive genes in the ripening process, we selected *SlGASA1* for further study as it was the only *SlGASA* family member induced by GA but repressed by PAC and was downregulated during ripening. Subcellular localization performed in *Nicotiana benthamiana* leaf protoplasts showed that SlGASA1 localizes to both the cytoplasm and the nucleus (Fig. 3A). To explore whether *SlGASA1* is regulated by ethylene, we examined the effect of treating mature green (MG) tomato fruit with ethylene or 1-MCP (1-methylcyclopropene, an ethylene perception inhibitor) on the transcript levels of this gene by RT-qPCR; transcripts of the ethylene-induced gene *E4* were used as a control. The transcript accumulation of *SlGASA1* was repressed by ethylene but induced by 1-MCP, suggesting a negative role of SlGASA1 in fruit ripening (Fig. 3B).

### Overexpression of *SlGASA1* in tomato leads to a delay in fruit ripening

**Figure 4:**
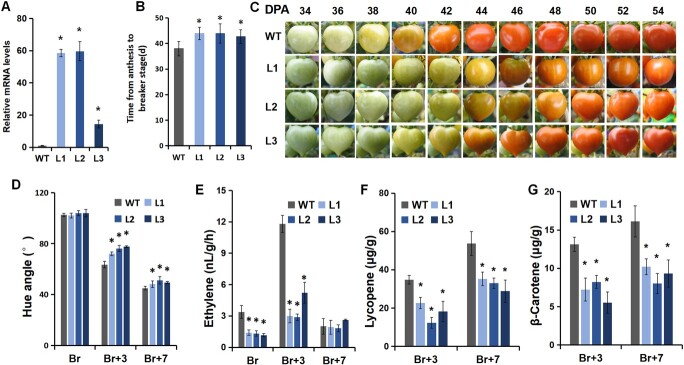
Overexpression of *SlGASA1* delays fruit ripening. **A***SlGASA1* relative expression levels in *SlGASA1-*OE breaker stage fruits. L1, L2, and L3 represent three independent *SlGASA1-*OE lines. ^*^P < 0.05 (Student’s *t*-test). **B** Time period from anthesis to fruit reaching the breaker stage in WT and the three *SlGASA1-*OE lines. Values represent means ± SD. ^*^P < 0.05 (Student’s *t*-test). **C** Fruit ripening process of WT and *SlGASA1-*OE lines. DPA, days post anthesis. **D** Hue angle value of WT and *SlGAST1-*OE fruits during fruit ripening. **E** Ethylene production in WT and *SlGAST1-*OE fruits during fruit ripening stages. **F** and **G** The content of lycopene and β-carotene in WT and *SlGAST1-OE* fruits at two ripening stages (Br + 3 and Br + 7). L1, L2, and L3 are three independent *SlGAST1-*OE lines. ^*^P < 0.05 (Student’s *t*-test).

Because *SlGASA1* expression was significantly downregulated at the ripening initiation stage (i.e. breaker stage), we generated *SlGASA1* overexpression (*SlGASA1*-OE) lines using a fruit ripening-specific promoter (*E8*) to investigate the role of *SlGASA1* in fruit ripening. We obtained more than 10 independent transgenic lines and performed RT-qPCR to assess the relative accumulation of *SlGASA1* transcripts in breaker stage fruits. Three independent T_2_ lines (L1, L2, L3) that showed significantly higher *SlGASA1* transcript levels (in the range 15–60-fold) compared to the wild type (WT) were selected for physiological and molecular analyses (Fig. 4A).

The *SlGASA1*-OE plants showed a significant delay in the onset of ripening compared to the WT (Fig. 4B). Under our cultivation conditions, the average time from anthesis to the breaker stage in WT fruits was approximately 38 days (Fig. 4C), while for *SlGASA1*-OE fruits, the average time was 43 (L1), 43 (L2), and 42 (L3) days (Fig. 4C). The color change measurements (hue angle values) confirmed the delayed ripening of *SlGASA1*-OE fruits compared to the WT after the breaker stages (Fig. 4D). We also monitored ethylene emission during ripening, finding that ethylene production is significantly reduced at the breaker (Br) and breaker+3 stages (Br + 3) in fruits of the three *SlGASA1*-OE lines (Fig. 4E). These results indicate that in addition to a delay in ripening initiation, fruit ripening after the breaker stage is also delayed in the *SlGASA1*-OE lines.

Carotenoid accumulation is an important parameter indicating the progress of ripening. To further investigate the influence of *SlGASA1* overexpression on fruit ripening, we measured the carotenoid contents in the WT and *SlGASA1*-OE lines. The contents of lycopene (red) and β-carotene (orange-yellow), two critical carotenoid components responsible for tomato color, were significantly reduced in *SlGASA1*-OE fruits after the breaker stage (Fig. 4F and G). These results further support the finding that ripening is delayed in *SlGASA1*-OE fruits, and they point to a repressive role for SlGASA1 in tomato fruit ripening.

### Expression of ripening-related genes is suppressed in *SlGASA1*-OE fruits

**Figure 5:**
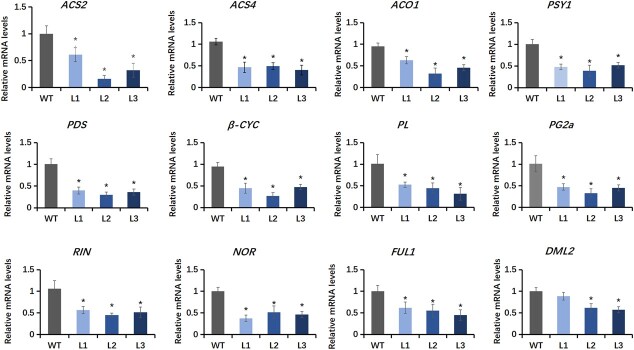
Relative expression of ripening-related genes in wild-type and *SlGASA1-*OE fruits at Br stage. The relative expression levels of each ripening gene in WT were normalized to 1, with the *SlActin* used as an internal control. Values represent means ± SD from three biological replicates. L1, L2, and L3 are three *SlGASA1-*OE lines. ^*^P < 0.05 (Student’s *t*-test).

The ripening-delayed phenotype prompted us to examine the expression levels of ripening-associated genes in *SlGASA1*-OE lines. In line with the delayed ripening and decreased ethylene production of these lines, the transcript levels of *ACS2*, *ACS4*, and *ACO1*, three key ethylene biosynthesis genes, were significantly reduced in *SlGASA1*-OE fruits compared to the WT at the breaker stage (Fig. 5). Moreover, the transcript levels of *PSY1*, *PDS*, and*β-CYC*, encoding critical regulators of flux through the carotenoid pathway, were reduced in *SlGASA1-*OE fruits (Fig. 5), which is consistent with their decreased carotenoid accumulation. Furthermore, the transcript levels of genes associated with cell wall modification and fruit softening, such as *PG2a* (*POLYGALACTURONASE 2a*) and *PL* (*PECTATE LYASE*)*,* were also significantly lower in *SlGASA1-*OE fruits than in WT at the breaker stage (Fig. 5). In addition, the expression levels of *RIN*, *NOR*, and *FUL1*, three key ripening regulators [42], were significantly downregulated in the *SlGASA1*-OE lines (Fig. 5). Interestingly, the relative transcript levels of *DEMETER-LIKE PROTEIN 2* (*DML2*), encoding a key DNA demethylase that controls fruit ripening in tomato, were also lower in L2 and L3 compared to the WT (Fig. 5). These results further support the repressive role of SlGASA1 in regulating fruit ripening in tomato*.*

### Silencing of *SlGASA1* leads to accelerated fruit ripening

**Figure 6:**
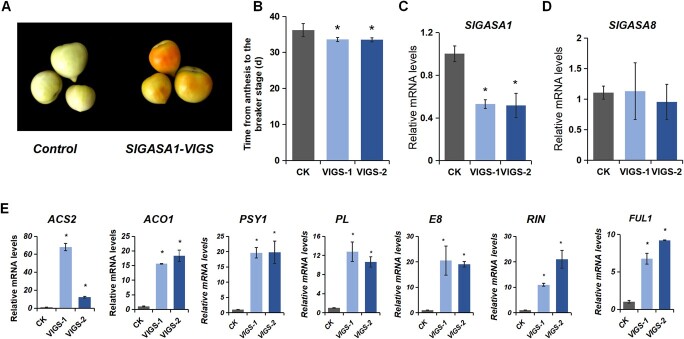
Downregulation of *SlGAST1* accelerates fruit ripening in tomato. **A** Downregulation of *SlGASA1* results in accelerated fruit ripening. WT fruits were injected with an Agrobacterium culture containing the pTRV2 or pTRV2-SlGASA1 construct at 30 DPA (days post anthesis) and photographed at 37 DPA. **B** Time from anthesis to fruit reaching the breaker stage in control and *SlGASA1*-VIGS fruits. VIGS-1 and 2 are two batches of treated fruits. **C** and **D** Relative expression levels of *SlGASA1* and *SlGASA8* in control (CK) and *SlGASA1-*VIGS lines. ^*^P < 0.05 (Student’s *t*-test). **E** Relative expression levels of the key ripening-related genes *ACS2, ACS4*, *PSY1*, *PL*, *E8*, *RIN,* and *FUL1* in control (CK) and *SlGASA1*-VIGS-treated fruits. Values represent means ± SD of three biological replicates. ^*^P < 0.05 (Student’s *t*-test).

To gain more insight into the role of SlGASA1 in fruit ripening, we used virus-induced gene silencing (VIGS) to knockdown *SlGASA1* transcript levels in tomato fruit. Accordingly, we infiltrated WT fruits at 30 DPA with Agrobacterium (*Agrobacterium tumefaciens*) harboring either pTRV2-SlGASA1 or pTRV2 (as the control). The *SlGASA1*-silenced fruits exhibited earlier ripening than the control (Fig. 6A). In control fruits, the average time from anthesis to the breaker stage was approximately 37 days, whereas *SlGASA1*-silenced fruits reached the breaker stage at 33 DPA (Fig. 6B). RT-qPCR analysis showed that *SlGASA1* transcript levels are significantly lower in *SlGASA1*-silenced lines compared to the control (Fig. 6C). By contrast, the expression levels of *SlGASA8*, a family member closely related to *SlGASA1*, displayed no significant change in *SlGASA1*-silenced lines (Fig. 6D), suggesting that the VIGS construct is specific for *SlGASA1*. In line with their accelerated fruit ripening, the transcript levels of ripening-associated genes including *ACS2, ACO1, PSY1, PL, E8, RIN,* and *FUL1* were significantly higher in *SlGASA1*-silenced fruits than those of the control at 37 DPA (Fig. 6E). These results further support the notion that SlGASA1 represses fruit ripening.

### SlGASA1 interacts with the ripening regulator FUL1 and represses its upregulation of *ACS2* and *ACO1*

**Figure 7:**
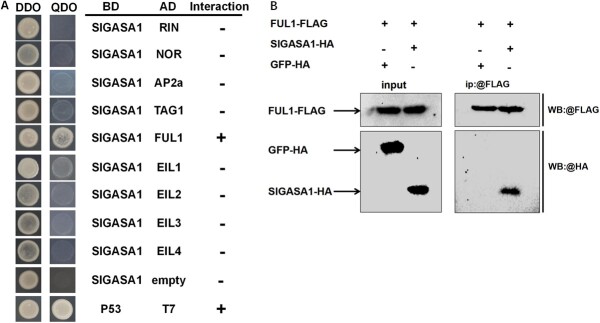
SlGASA1 interacts with FUL1 both *in vitro* and *in vivo*. **A** Screening for ripening regulators that interact with SlGASA1 by yeast two-hybrid. SlGASA1 protein was fused to the GAL4 DNA-binding domain as bait, and RIN, NOR, AP2a, TAG1, FUL1, and EIL1–4 were fused with the GAL4 activation domain as preys. Interactions between P53 and T7 and between SlGASA1-BD and empty AD were used as positive and negative controls, respectively. **B** Co-immunoprecipitation assay validating the interaction of SlGASA1 and FUL1. *SlGAST1-HA* and *FUL1-FLAG*, *FUL1-FLAG* and *GFP-HA* were transiently co-infiltrated in *N. benthamiana* leaves as described in the ‘Materials and methods’ section.

To explore the possible mechanism underlying the role of SlGASA1 in regulating fruit ripening, we examined the interaction between SlGASA1 and a set of ripening regulators: RIN, NOR, AP2a, TAG1, FUL1, and EIL1–4 (ETHYLENE-INSENSITIVE3-LIKE 1–4) by performing yeast two-hybrid assays. SlGASA1 interacted with FUL1 (Fig. 7A), a key MADS-box transcription factor in the fruit ripening regulatory network, but not with any of the other transcription factors tested. To validate the interaction between SlGASA1 and FUL1 *in vivo*, we conducted co-immunoprecipitation (Co-IP) assays in *N. benthamiana* leaves. SlGASA1-HA co-precipitated with FUL1-FLAG, but not with GFP-FLAG, following immunoprecipitation with an anti-FLAG antibody (Fig. 7B). These results indicate that SlGASA1 and FUL1 interact both *in vitro* and *in vivo*.

**Figure 8:**
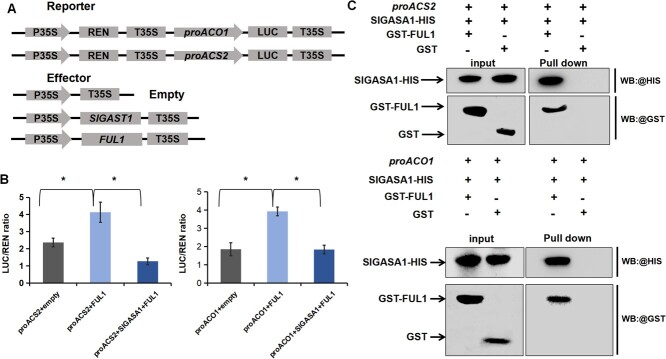
SlGASA1 represses the activation of ethylene biosynthesis genes by FUL1. **A** A schematic illustration of the *LUC* reporter and effector constructs used in transient expression assays. **B** SlGASA1 represses the activation of *ACS2* and *ACO1* by FUL1. Each value represents the mean of three biological replicates, and vertical bars represent SD. LUC, firefly luciferase; REN, Renilla luciferase. ^*^P < 0.05 (Student’s *t*-test). **C** Binding of SlGASA1 and FUL1 to the *ACS2* and *ACO1* promoters. A DNA pull-down assay showed that SlGASA1 and FUL1 bind to the ACrG box region in the *ACO1* or *ACS2* promoter. GST-FUL1, SlGASA1-HIS, and GST proteins were produced and purified in *Escherichia coli*. SlGASA1-HIS and GST-FUL1, SlGASA1-HIS and GST were incubated with *ACO1* or *ACS2* promoter probes, and the interactions were detected with anti-GST antibody.

Because both *ACS2* and *ACO1*, which are key ethylene biosynthesis genes controlling climacteric fruit ripening, are direct targets of FUL1 and were significantly downregulated in *SlGASA1*-OE fruits but upregulated in *SlGASA1*-silenced fruits, we reasoned that the interaction between SlGASA1 and FUL1 might affect the transcriptional regulation of *ACS2* and *ACO1* by FUL1. To test our hypothesis, we performed dual-luciferase reporter assays in *N. benthamiana* leaves (Fig. 8A). Transient expression of *FUL1* in *N. benthamiana* leaves enhanced the promoter activity of *ACS2* and *ACO1*, but co-expression of *FUL1* and *SlGASA1* significantly reduced the transcriptional activation of these two genes (Fig. 8B). To examine whether SlGASA1 recruits FUL1 to the *ACS2* and *ACO1* promoters, we performed DNA pull-down assays with biotin-labeled *ACS2* and *ACO1* promoters. The *ACS2* or *ACO1* promoter pulled down SlGASA1 only in the presence of FUL1 (Fig. 8C), suggesting that SlGASA1 and FUL1 form a complex to repress the transcription of *ACS2* and *ACO1*. Taken together, these results suggest that SlGASA1 regulates fruit ripening by repressing the transcriptional regulation of key ripening-associated genes by FUL1.

## Discussion

GA plays a negative role in fruit ripening in tomato [40, 43], but the roles of GA-responsive genes in fruit ripening remain largely unknown. Here, by identifying *GASA* family genes in tomato, we revealed that *SlGASA1*, a GA-responsive gene with low expression after the breaker stage, encodes a repressor of fruit ripening that represses the transcriptional activation of the ethylene biosynthesis genes *ACS2* and *ACO1* by FUL1. Our findings of the role and mode of action of SlGASA1 in fruit ripening provide new insights into the regulatory mechanism of climacteric fruit ripening, and they extend our understanding of the roles of GASA family members in various developmental processes.

*GASA* family genes have been identified in a number of plant species, and different family genes have been found to be involved a wide range of developmental processes, including cell elongation [16, 17, 21], the floral transition [17, 28], fruit and seed development [13, 21, 28], defense against pathogens [5–8], and resistance to abiotic stress [6]. Among the 17 *SlGASA* family genes, genes in subgroup II were specifically expressed in flower buds (Fig. 2B; Fig. S4, see online supplementary material), suggesting that they might function in the floral transition. Subgroup IV genes were mainly expressed in roots and fruits during early development (Fig. 2B; Fig. S4, see online supplementary material), supporting the involvement of these subgroup members in root and fruit development. Interestingly, in contrast to the high expression levels of the two *SlGASA* genes in subgroup I, the four *SlGASA* genes in subgroup III were expressed at low levels after the onset of ripening (Fig. 2B; Fig. S4, see online supplementary material), suggesting that they play a negative role in fruit ripening. Notably, the four genes in subgroup III, which were expressed at low levels during ripening, were highly expressed in vegetative and reproductive tissues, suggesting that they play important roles in vegetative and reproductive development. All these expression patterns indicate that *GASA* family genes play conserved roles in different plants. However, since most GASA family members have not been functionally characterized, future work should focus on revealing the roles and underlying regulatory mechanisms of *GASA* family genes during different developmental processes and stress responses.

Although *GASA* family genes were shown to be involved in a wide range of developmental process, the functions of *GASA* genes in fruit ripening remain unknown. In addition to tomato, *GASA* family members have been identified in strawberry and apple, which also have fleshy fruits. In strawberry, two *FaGASA* family genes, *FaGAST1* and *FaGAST2*, were associated with fruit ripening based on their expression during fruit ripening [20, 21]. However, their functions were reported to be related to fruit size determination [20, 21], and whether they are involved in fruit ripening remains elusive. Several *MdGASA* family members in apple, such as *MdGASA3*, *MdGASA13*, and *MdGASA26*, are also highly expressed in fruits [12], but their functional significance in fruit ripening remains unclear. Thus, our demonstration of the role and mode of action of SlGASA1 in fruit ripening in tomato extends our knowledge of the functions of GASA family members in fleshy fruits. Nonetheless, in addition to SlGASA1, the roles and underlying regulatory mechanisms of other SlGASA family members in fruit ripening requires further investigation, as several *SlGASA* genes displayed ripening-associated expression patterns, including *SlGASA13* and *SlGASA17*, whose transcripts accumulated to high levels during ripening.

Identifying ripening regulatory genes and revealing their underlying mechanisms represent important steps toward dissecting the ripening regulatory networks of fruits. Our study identified a novel ripening gene and revealed the underlying mechanism of the encoded protein in regulating fruit ripening. Like most reported regulators that mediate ripening processes in an ethylene-dependent manner [33, 34, 42, 44], SlGASA1 also regulates the ripening of tomato fruit by affecting ethylene biosynthesis. Interestingly, our results showed that SlGASA1 is unable to directly bind to the promoters of the ethylene biosynthesis genes *ACS2* and *ACO1*. Instead, it recruits the key ripening transcription factor FUL1 and represses the activation of *ACS2* and *ACO1* transcription by FUL1. Our findings reveal a novel regulatory mechanism controlling fruit ripening. However, the molecular mechanisms underlying how the interaction between SlGASA1 and FUL1 represses the transcription of ethylene biosynthesis genes require further investigation.

## Materials and methods

### Data collection and tomato *GASA* family identification

Protein sequences of tomato GAST1 (Solyc02g089350) and GASAs in Arabidopsis were obtained from the database of NCBI (http://www.ncbi.nlm.nih.gov/). The tomato (SL4.0) reference genome sequence and annotations were obtained from the SGN database (https://solgenomics.net/).

Based on studies of GASA proteins in Arabidopsis, the conserved domain of GASA proteins was identified as the GASA domain (PF02704). We preliminarily identified 20 putative GASA proteins encoded in the tomato genome using a Hidden Markov model (HMM). We manually determined whether these putative GASA proteins contained a cleavable signal peptide in their N termini, ultimately characterizing 17 GASA proteins in tomato. In addition to Solyc02g089350 (*SlGASA1*), we named the 16 remaining *SlGASA* genes in tomato based on their chromosomal positions. The basic information including molecular weight (Mw) and isoelectric point (pI) of all SlGASA proteins were obtained by the online program Expasy.

### Protein sequence alignment and phylogenetic tree construction

The alignment of the protein sequences of AtGASAs in Arabidopsis, OsGASAs in rice and SlGASAs in tomato was performed using MEGA X. The bad alignment regions of all protein sequences participating in topology structure construction were removed by trimAl tool [45]. A phylogenetic tree was then generated by the method of maximum-likelihood (ML) with poisson correction and 1000 bootstrap replicates in IQ-TREE [46]. The visualization of the phylogenetic tree was performed using Evolview (www.evolgenius.info/). We also built a phylogenetic tree using the sequences of tomato SlGASA proteins alone, and the results were consistent with the results obtained using the previous approach. The protein sequences of SlGASA family are provided in Data S2 (see online supplementary material).

### Analyses of conserved motifs, conserved domains, and *cis*-acting elements in SlGASA promoters

The analysis of the conserved motifs of SlGASA proteins were performed by the program of MEME (5.05) (http://meme.nbcr.net/meme/), and the conserved domains of tomato GASA proteins were predicted using Pfam (http://pfam.xfam.org/). The upstream 2.0 kb genomic DNA sequences from the translation initiation codon (ATG) were taken as the promoter regions of *SlGASAs*. The *cis*-elements of the *SlGASA* promoter regions were detected by PlantCare with default parameters. Phytohormone-related *cis*-elements are summarized in Data S1 (see online supplementary material). The visualization of the relevant data was conducted using TBTools [4].

### Plasmid construction and tomato genetic transformation

To generate the *E8:SlGASA1*-pBin19 construct, the coding sequence of *SlGASA1* was amplified and cloned into the vector of pDONR207, followed by mobilization into the Gateway vector pBin19. The primers used for construction of *E8:SlGASA1*-pBin19 are listed in Data S3 (see online supplementary material). The tomato genetic transformation mediated by Agrobacterium (*A. tumefaciens*) was performed as described previously [47]. Half-strength Murashige and Skoog medium with kanamycin (100 mg L^−1^) was used to select the transgenic tomato lines.

### Subcellular localization

To generate the *35S:SlGASA1:GFP* construct, we amplified the *SlGASA1* coding sequence excluded the stop codon and inserted into a vector harboring *35S:GFP*. The *35S:SlGASA1:GFP* construct and the empty *35S:GFP* vector were then transferred into *Nicotiana benthamiana* leaf protoplasts as previously described [48]. Following culture in the dark for 22 hours, the subcellular location was observed under a confocal laser scanning microscope.

### Tomato growing conditions

The wild type and transgenic tomato plants (*S. lycopersicum* L. cv Micro-Tom) used in this study were grown in a greenhouse with the conditions descried in Deng *et al*. [49].

### VIGS experiments

Regarding the VIGS experiments, WT tomato fruits were collected at 30 DPA and injected with 100 μL Agrobacterium culture containing the pTRV2-SlGASA1 construct or pTRV2 (control) into fruit tissues through the calyx scar. Seven days after injection, fruit pericarp samples were frozen by liquid nitrogen and stored at −80°C.

### RNA isolation and RT-qPCR

Tomato pericarp samples for RT-qPCR analysis were collected at different growth and developmental stages. A plant RNA extraction kit (BIOFIT, Chengdu, China) was used to isolate the total RNA from different tomato tissues. The procedure including removal of genomic DNA, cDNA generation, and quantitative PCR (qPCR) analysis were performed as described in Deng *et al*. [49]. The sequences of primers used for RT-qPCR are provided in Data S3 (see online supplementary material).

### Transcriptome profiling

The RNA-seq data used for transcriptomic analysis were obtained from our previous publication [41] which is available in the Big Data Center’s Genome Sequence Archive (http://bigd.big.ac.cn/) under accession numbers CRA001723 and CRA001712. Transcriptome profiling was conducted as described in our previous article [41], clean reads were mapped to the tomato reference genome (version 4.0) using HISAT2 [50] and then normalized to reads TPM (transcript per million).

### Phytohormone treatments

To treat fruits with GA_3_ or the GA biosynthetic inhibitor paclobutrazol (PAC), WT tomato fruits were collected at 32 DPA and injected through the calyx scar with 100 ppm GA_3_, 100 ppm PAC or distilled water (control) using a syringe. After 2 days, a group of GA_3_-treated fruits was treated with 100 ppm ethylene for 24 hours in a sealed jar; 20 fruits were used per treatment. The treated fruit samples were then stored at −80°C until use.

For the treatments of ethylene and 1-MCP, WT fruits at the MG stage were treated with ethylene (100 ppm) for 12 hours or 1-MCP (40 ppm) for 16 hours in sealed jars. Treated fruit samples were stored at −80°C.

### Measurement of fruit color

WT and transgenic tomato fruits were collected at different ripening stages. Their color measurement was performed as described by Deng *et al*. [51].

### Measurement of ethylene content

WT and transgenic tomato fruits at different ripening stages were picked and incubated at room temperature for 2 hours in open 150-mL jars. Each jar with a single fruit were sealed and then kept for 2 hours at room temperature, and 1 mL gas samples from headspace were collected with a syringe and analysed using an Agilent 7890B gas chromatograph. Ethylene content was quantified as describe in Deng *et al*. [49].

### Carotenoid and chlorophyll measurements

WT and transgenic tomato fruits were collected at different ripening stages, and the extraction and measurement of carotenoid were conducted as described in Deng *et al*. [51].

### Yeast two-hybrid assay

The full-length coding sequences of *RIN*, *NOR*, *EIL1–4*, *TAG1*, and *FUL1* were cloned into pGADT7 for generation of the prey constructs. The coding sequence of *SlGASA1* was inserted into pGBKT7 as the bait construct. Yeast AH109 cells were co-transformed with different combination of the bait and prey constructs and grown on SD (synthetic defined) medium without Leu and Trp (SD-Leu-Trp) for 2–4 days. Interactions were tested based on the growth of yeast cultures on selection medium (SD-Leu-Trp-His-Ade).

### Dual-luciferase reporter assay

The vectors used for this transient transactivation assay were generated according to the GoldenBraid2.0 cloning strategy. Constructs of 35S:SlGASA1 and 35S:FUL1 were used as effectors, while promoter sequences of *ACO1* and *ACS2* were individually cloned upstream of the *Luciferase* (*LUC*) coding sequence to serve as the reporters. *Renilla* luciferase gene was used as an internal control. The Dual-Luciferase reporter assay system (Promega, Madison, WI, USA) was used to test the ratio of LUC to REN.

### Co-IP assay

The CDS of *SlGASA1* or *FUL1* was cloned into the pBTEX-FLAG or pBTEX-HA vector to generate the *FUL1-FLAG* or SlGASA1*-HA* overexpression construct, respectively. SlGASA1*-HA* and *FUL1-FLAG* were co-expressed in *N. benthamiana* leaves through infiltration mediated by Agrobacterium, and *FUL1-FLAG* and *FUL1-FLAG* were co-infiltrated in *N. benthamiana* leaves to serve as a control. Total proteins were obtained from the *N. benthamiana* leaves at 48–72 hours after Agrobacterium infiltration and incubated with anti-FLAG M2 magnetic beads (Sigma, M8823) for 12–16 h at 4°C. The isolated proteins were examined by immunoblot analysis using anti-HA (CST, #2367) and anti-FLAG (CST #14793) antibodies.

### DNA pull-down assay

Full-length *FUL1* and *SlGASA1* cDNAs were inserted into the pGEX-4 T-1 or pET-28a vector. Recombinant GST, GST-FUL1, and SlGASA1-HIS were produced in *Escherichia coli* strain BL21. Purification was performed using GST-tag Purification Resin (BeyoGold, P2262) and HIS-tag Purification Resin (BeyoGold, P2233). Primers used here can be found in Data S3 (see online supplementary material). The probes for the *ACS2* and *ACO1* promoters were produced by PCR with 5′-biotin-labeled primers. Purified SlGASA1-HIS was incubated overnight with 1.0 μg of biotin-labeled probe with GST or GST-FUL1 in HKMG buffer [100-mM KCl, 10 mM HEPES, 5 mM MgCl_2_, 1 mM DTT, 10% (v/v) glycerol, and 0.5% (v/v) NP-40, pH 7.9] containing protease and phosphatase inhibitors. Streptavidin-agarose beads were used to precipitate the biotin-labeled probes and proteins binding to the probes. The precipitated proteins were examined by immunoblot analysis using anti-GST (BeyoGold, AG768) and anti-HIS (BeyoGold, AH367) antibodies.

## Acknowledgements

This research was supported by the National Natural Science Foundation of China (32172643 and 32172271), the Applied Basic Research Category of Science and Technology Program of Sichuan Province (2021YFQ0071; 2022YFSY0059), and the Technology Innovation and Application Development Program of Chongqing (cstc2021jscx-cylhX0001). This project was also supported by the Fundamental Research Funds for the Central Universities (SCU2021D006).

## Author contributions

M.L. planned and designed the research; D.S., Z.Y., Y.L., and Y.Z. performed the experiments; K.L., Y.Z., Y.W., H.., X.Z., and H.C. analysed data; M.L. and H.D. wrote the manuscript, and D.G. helped improve the manuscript..

## Data availability

The authors confirm that all the experimental data are available and accessible via the main text and/or the supplemental data.

## Conflict of interests

The authors declare no conflict of interest.

## Supplementary data

Supplementary data is available at *Horticulture Research* online.

## Supplementary Material

Web_Material_uhac222
