# Supplementary material for: Genome-wide characterization of the tomato GASA family identifies SlGASA1 as a repressor of fruit ripening
Source: Hortic Res. 2022 Sep 28;10(1):uhac222. doi: 10.1093/hr/uhac222 (PMC9832878; doi:10.1093/hr/uhac222)
Supplement: Web_Material_uhac222 [file web_material_uhac222.zip › Supplemental Figures 1-5.pdf]

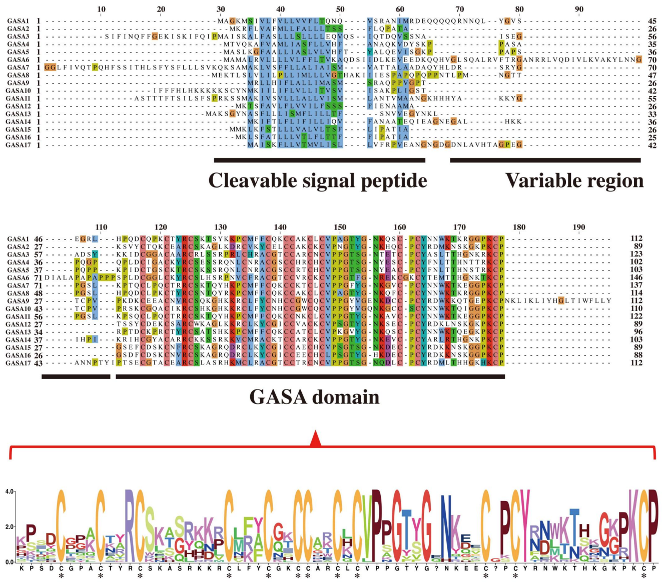

Fig.S1 Alignment of the protein sequences of GASA family in tomato. The protein sequences of SlGASAs possess a cleavable signal peptide at the N-terminus, about 18-29 amino acid residues, a variable region with polar amino acid residues, and a conserved GASA domain at the C-terminus, which is ~60 amino acids, including 12 cysteines.

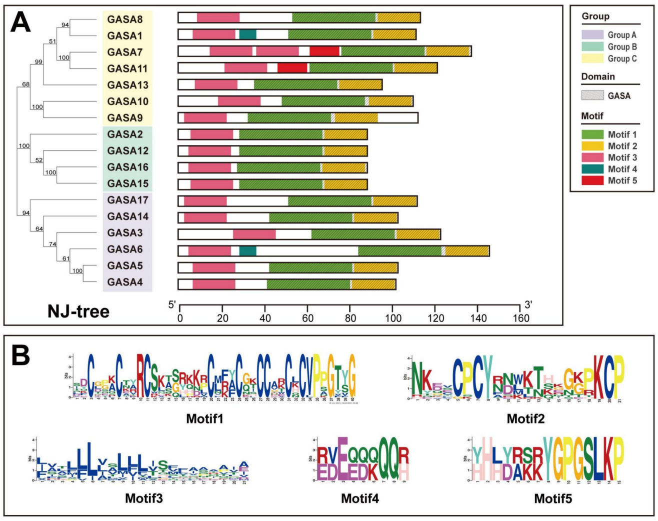

Fig. S2 Conserved domains and motifs analyses of tomato GASA family members. (A) Schematic representation of motifs and domain in SlGASA proteins. Different motifs are indicated by different colors. (B) The motif sequence logos of SlGASA proteins. Motif logo represents the amino acid composition of each motif.

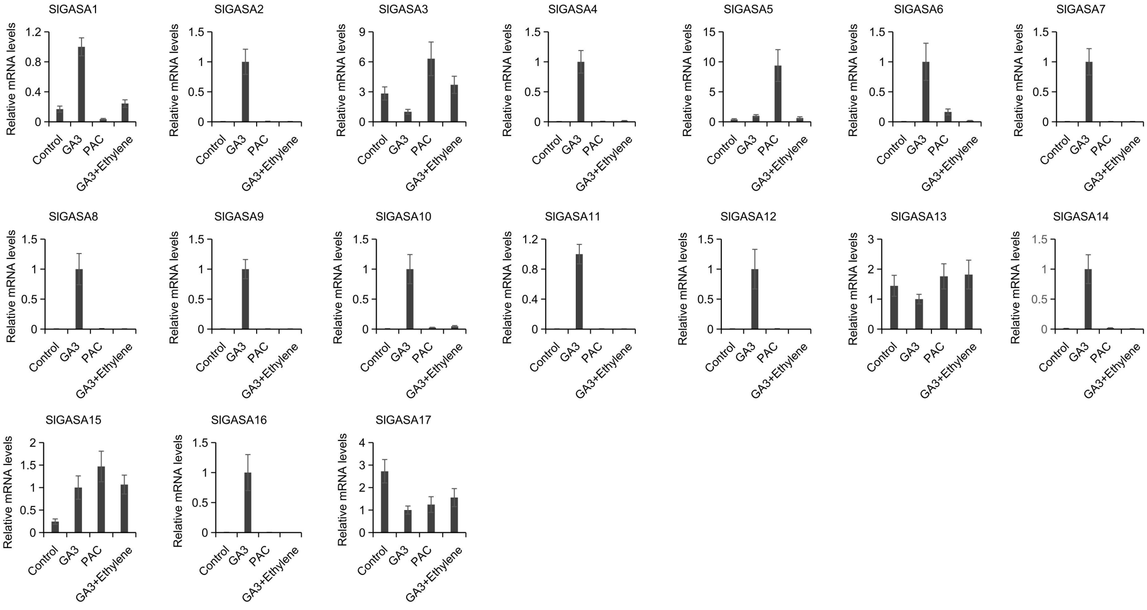

Fig. S3 Responsiveness of SlGASAs to GA, PAC (paclobutrazol), and GA3+Ethylene. RT-qPCR was performed after the treatment of GA3, PAC, and GA3+ethylene as described in the Material and Method.

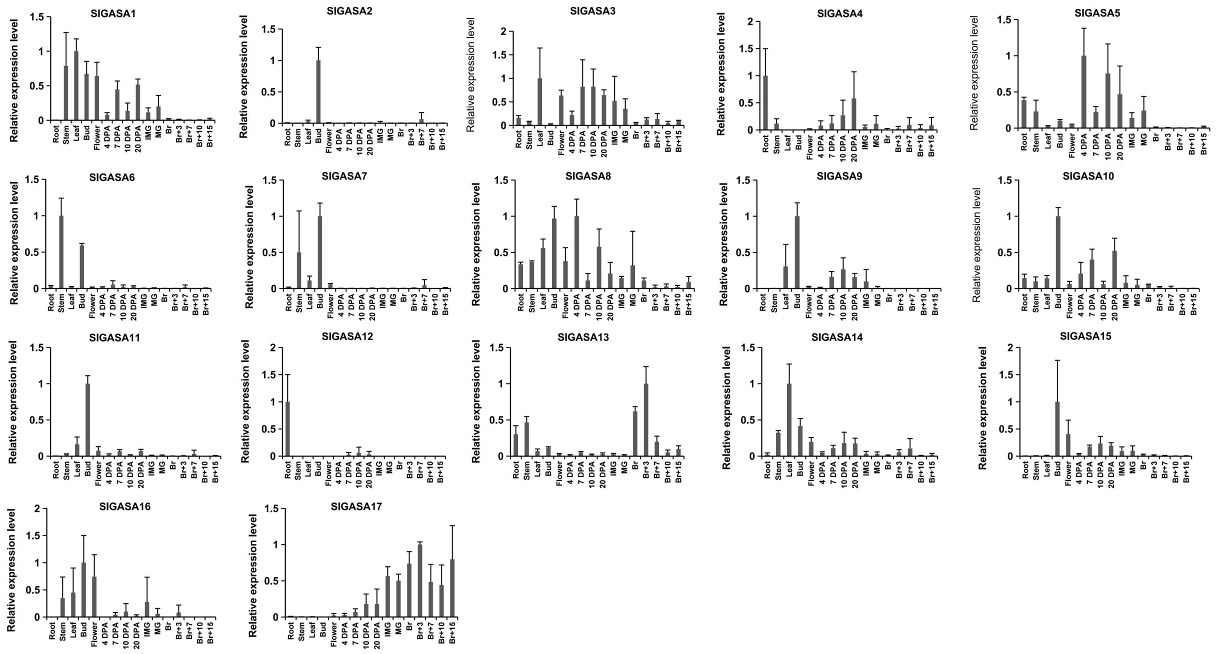

Fig. S4 Expression of SlGASAs in different tomato tissues and developmental stages. 7 DPA, fruit at 7 days after anthesis stage; 10 DPA, fruit at 10 days after anthesis stage; 20 DPA, fruit at 20 days after anthesis stage; IMG, immature green fruit; MG, mature green stage fruit; Br, breaker stage fruit; Br+3, fruit at 3 days post-breaker stage; Br+5, 5 days post-breaker; Br+7, 7 days post-breaker; Br+10, 10-day post-breaker; Br+15, 15 days post-breaker).

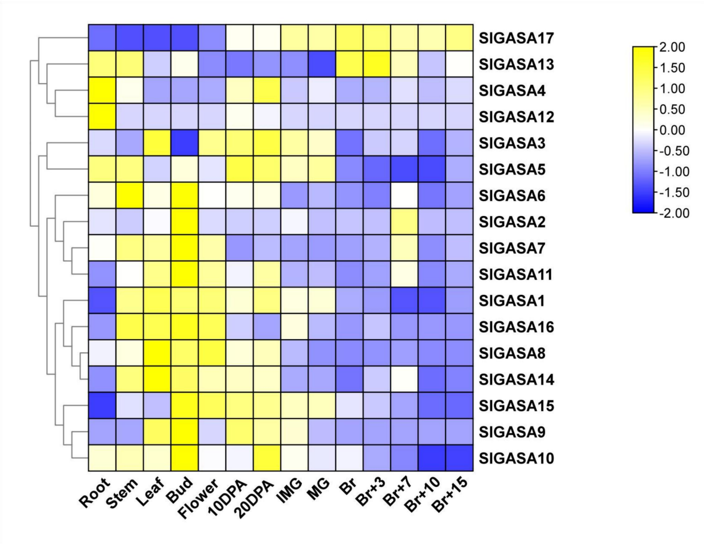

Fig. S5 Heatmap representation of the expression pattern of tomato GASA family genes in different tissues and developmental stages. The expression data was obtained from the RNA-seq project of Li et al., (2020). 10 DPA, fruit at 10 days after anthesis stage; 20 DPA, fruit at 20 days after anthesis stage; IMG, immature green fruit; MG, mature green stage fruit; Br, breaker stage fruit; Br+3, fruit at 3 days post-breaker stage; Br+5, 5 days post-breaker; Br+7, 7 days post-breaker; Br+10, 10-day post-breaker; Br+15, 15 days post-breaker).

.
